# Supplementary figures and images for: Reproductive biology of wild and domesticated Ensete ventricosum: Further evidence for maintenance of sexual reproductive capacity in a vegetatively propagated perennial crop
Source: Plant Biol (Stuttg). 2022 Feb 8;24(3):482–91. doi: 10.1111/plb.13390 (PMC9303740; doi:10.1111/plb.13390)

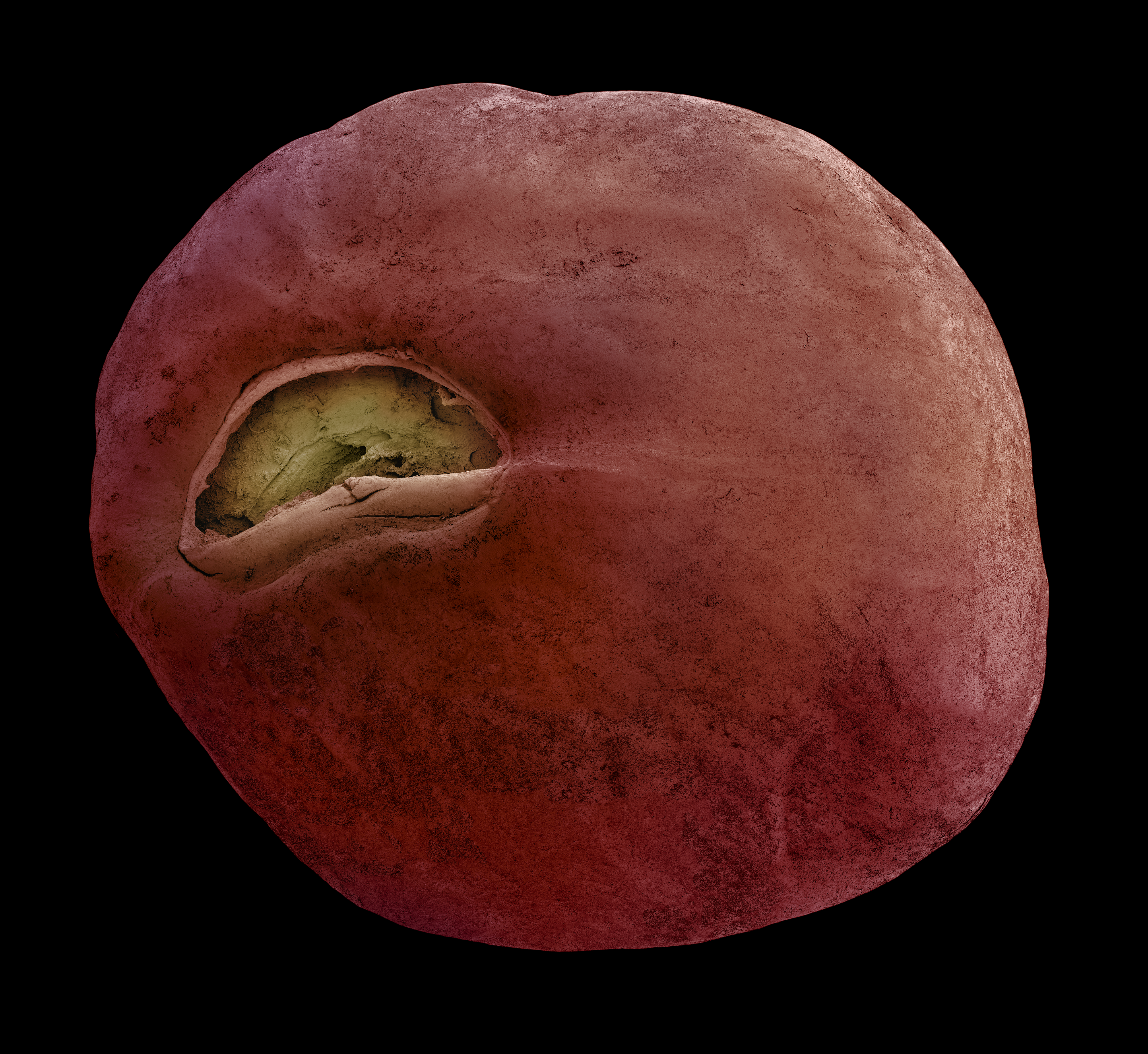

Supplement: Supplementary file 1 — Figure S1. X‐ray images of Ensete ventricosum seeds. [file PLB-24-482-s001.tif]
